# A cluster randomized controlled trial to assess the impact of SAFE on spousal violence against women and girls in slums of Dhaka, Bangladesh

**DOI:** 10.1371/journal.pone.0198926

**Published:** 2018-06-14

**Authors:** Ruchira Tabassum Naved, Mahfuz Al Mamun, Sanjida Akhter Mourin, Kausar Parvin

**Affiliations:** Health Systems and Population Studies Division, icddr,b, Dhaka, Bangladesh; University of Westminster, UNITED KINGDOM

## Abstract

**Background:**

Bangladesh reports one of the highest rates of intimate partner violence (IPV) in the world. Despite wide recognition of IPV as an important public health and human rights issue, evidence for IPV prevention is still inadequate. Lack of guidance on effective IPV prevention in Bangladesh resulted in targeting only women in most of the programmes.

**Methods:**

This paper assesses impact of SAFE, a 20-month intervention (March 2012 to October 2013) in slums of Dhaka on IPV and tests effectiveness of female only groups vs. no groups; and female + male groups vs. female only groups on IPV in the community using a three-arm cluster randomized controlled trial. SAFE’s core activities included interactive group sessions, community mobilisation, and services. The last two activities were common across arms.

**Findings:**

Regression analyses (female survey: baseline n = 2,666; endline n = 2,670) showed no effect of SAFE on IPV against women aged 15–29. However, sub-group analyses demonstrated 21% risk reduction of physical IPV against adolescent girls aged 15–19 in the female + male group intervention arm. A consistent reduction in sexual violence was observed in both female and female + male arms for both groups of women, but the results were not statistically significant.

**Interpretation:**

The findings emphasise the importance of combining male and female interventions for reducing physical IPV against adolescent girls. Implications for future research have been discussed.

## Introduction

### Main challenges in reducing intimate partner violence

Levels of intimate partner violence (IPV) remain high in many countries of the world despite wide recognition of its multifaceted adverse consequences on individuals, families, communities and nations. Whilst the evidence-base of what works to prevent IPV is growing, it remains particularly limited in low and middle income countries inhibiting implementation of effective programmes and policies. Some interventions have been found promising in reducing IPV at the individual level [1–4]. The existing literature does not, however, provide clear guidance on how to reduce IPV at the community level [5]. To our knowledge, SASA! and SHARE implemented in Uganda are the only randomized controlled trials (RCT) in the developing world that assessed impact on IPV in the community. Reduction of physical and/or sexual IPV in SASA!, however, was not statistically significant [5], while SHARE found a statistically significant reduction in IPV at the community level [6].

A systematic review shows that economic empowerment of women combined with female and male training, and community mobilization generated promising results in preventing violence against women (VAW) [7]. Common features of effective interventions mentioned by this review include: participatory group sessions, engaging multiple stakeholders, promoting greater communication, shared decision making, and non-violent behaviour in intimate relationships. Using a cluster RCT this paper assesses community level effects of Growing up Safe and Healthy (SAFE) on IPV against women and girls. SAFE addressed sexual and reproductive health and rights (SRHR) and VAWG in Dhaka slums, drawing on existing evidence.

### The context

Bangladesh experiences very high rates of IPV. Approximately 54% of ever-married women reported lifetime physical and/or sexual IPV perpetrated by their husbands and 27% reported such IPV during the last 12 months. About 11% of ever married women reported economic IPV in their lifetime.^8^ Prevalence of physical IPV during the last 12 months in urban slums is the highest (35%) compared to other urban areas (20%) and rural Bangladesh (22%) [8–9] suggesting greater vulnerability of slum-dwelling women and girls to IPV.

Although there is important national legislation regarding VAW in Bangladesh [10–11], its implementation is rare. Many programmes target only a single tier of the society (e.g., individual or community) [12]. Despite wide recognition of the importance of targeting both men and women in IPV prevention efforts [1,13], only women are targeted in most interventions [12,14]. Finally, few interventions have been rigorously evaluated or documented, limiting opportunities to learn from past experiences. SAFE aimed to promote SRHR and address VAW was designed and implemented to address these gaps [15].

### Conceptual framework

**Fig 1:**
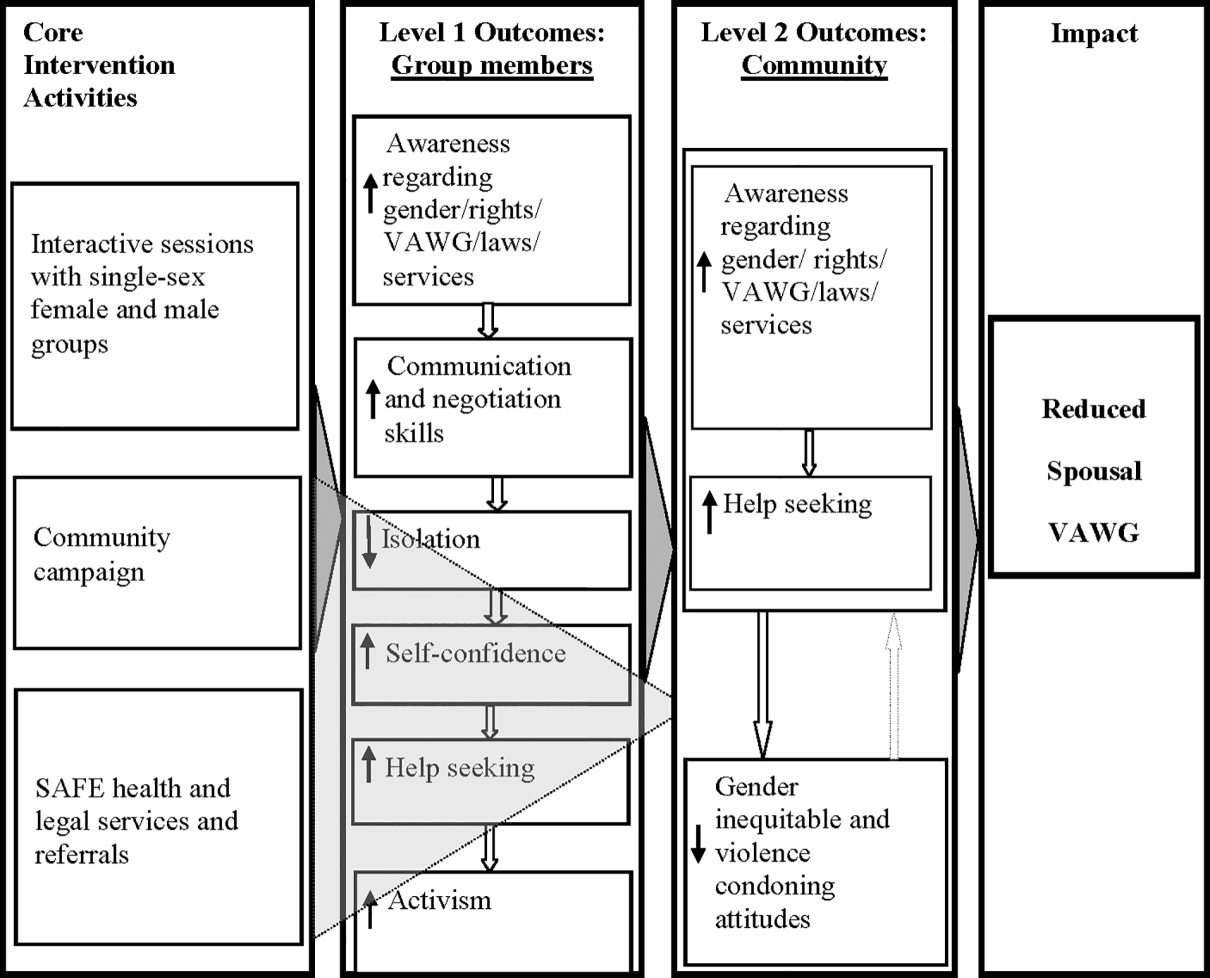
SAFE’s conceptual framework.

The theory of change underlying SAFE’s design considers a programme, involving different stakeholders as more effective than a siloed intervention [16–18]. SAFE hypothesized that its core interventions encompassing interactive group sessions [3] and community campaigns would promote awareness, gender equitable attitudes and activism (raising voices) for addressing violence (Fig 1) [7]. A related assumption was that group sessions would reduce isolation of abused women and enhance their self-confidence [19]. Group sessions were expected to strengthen the participants’ communication and negotiation skills and capacity to address IPV [20–22]. It was hypothesized that conducting group sessions with females and males would be much more effective than sessions with female groups only [1,13].

It was also expected that the knowledge, attitudes, and practices of SAFE group members would diffuse particularly through activism of the group members [21,23–24].

### Hypotheses

This paper focuses on impact of SAFE on IPV reduction in the community. The hypotheses of SAFE specific to IPV were:

H_1_: Interactive female group sessions on gender, health, rights, and life skills (F) will achieve a reduction in IPV in the community during the last 12 months compared to community mobilization and services only.
H_2_: Interactive gender segregated female and male group sessions on gender, health, rights, and life skills (F+M) will be more effective in reducing IPV in the community during the last 12 months than female only group intervention (F).

## Methods

### The intervention

SAFE is a multi-sectoral and multi-tier 20 month (March 2012 to October 2013) programme designed for females aged 10–29 and males aged 18–35 years by an icddr,b led consortium bringing together Bangladesh Legal Aid and Services Trust (BLAST), Nari Maitree (NM) and We Can Campaign (WCC), Marie Stopes Clinic (MSC), and Population Council (PC). Design of SAFE intervention was inspired by programmes such as Stepping Stones [17,20,25]. The study sites included 19 slums within 2km radius from three MSCs from Mohakhali, Mohammadpur, and Jatrabari in Dhaka city. Different partners brought different expertise to the project.

The Core activities of SAFE included: (1) Group sessions, (2) Community mobilization, (3) Health and legal services, and (4) Training and advocacy. The activities are briefly described below.

### Awareness-raising through group sessions

Due to limited power and isolation of women and girls in Bangladeshi society [26] it was deemed important to develop their social capital through female group formation. Based on evidence, interactive group sessions were supposed to address isolation, build confidence [19], and change attitudes and behaviours [20,27]. Group members were expected to raise voice and engage in activism [20,21–23,28].

Targeting men was considered important for the following reasons. First, according to the literature, IPV is higher in areas with high prevalence of gender inequitable [29] and violence condoning attitudes [30–32]. Second, young men are found to be amenable to change and with appropriate intervention they demonstrate the potential of becoming allies in the struggle against VAWG [1,13,25,33].

MSC staff formed a total of 600 (200 per site) SAFE groups (198 unmarried female; 252 married female; 75 unmarried male and 75 married male groups). Eligible participants (married and unmarried females aged 10–29; males aged 18–35) were identified using enumeration data. SAFE did not attempt to enrol couples in the groups. The average group size was 15. Eighteen eligible persons were recruited in each group expecting session attendance by 15. All group members were encouraged to participate in all the sessions. About 28% of the married women and 19% of the married men dropped out. Major reasons for dropout for both groups were outmigration (50% and 62% correspondingly) and time constraint (35% for both categories). About 8% married women mentioned restrictions from family and 1–2% females and males dropped out due to eviction. Dropouts were replaced by new members in the same age and marital status categories using enumeration data. At endline, the proportion of eligible community members covered by SAFE group intervention was 51% of females and 15% of males.

A total of 13 two-hour interactive sessions over a 20-month period were conducted separately with females and males. The sessions included games, breakout sessions for discussing and analysing issues, role plays, and presentation of short plays depicting relevant scenarios. Session topics were sequenced, starting with relatively less sensitive issues (gender and rights); gradually introducing more sensitive topics (SRHR, VAWG); and finishing with positive sessions on healthy relationship; life skills (i.e., interpersonal communication, negotiation and conflict resolution); and available sources of services. The training modules for female and male sessions were similar. Average session attendance was similar across gender (5.8 sessions for females; 5.7 sessions for males). As shown in Table 1 attendance was higher towards the final sessions for most of the groups.

**Table 1:** Number and percentage of participants attending each session.

Session	Female Married		Female Unmarried		Male Married		Male Unmarried	
	n	%	n	%	n	%	n	%
1: Gender, violence and life skills	3314	47.0	1870	46.9	980	44.6	956	48.7
2: Gender, violence and life skills review session	3187	45.2	1757	44.1	982	44.7	1038	52.9
3: Rights and marriage	2673	37.9	1442	36.2	699	31.8	774	39.4
4: Laws on violence against women	2580	36.6	1402	35.2	593	27.0	743	37.9
5: Review session	1311	18.6	1201	30.1	566	25.8	629	32.0
6: Sexual and reproductive health and rights (SRHR)	2549	36.2	1343	33.7	747	34.0	698	35.6
7: Growing up: physical and mental changes	2572	36.5	1387	34.8	654	29.8	650	33.1
8: Family planning and contraception	2378	33.7	1366	34.3	1045	47.5	900	45.8
9: Safe motherhood	3111	44.1	1683	42.2	1135	51.6	918	46.8
10: Reproductive tract infection and sexually transmitted diseases	3433	48.7	1817	45.6	1110	50.5	919	46.8
11: HIV/AIDS and healthy relationships	3490	49.5	1876	47.1	1037	47.2	940	47.9
12: Services and facilities	3717	52.7	1937	48.6	1024	46.6	943	48.0
13: Review Session	3979	56.5	2143	53.7	1160	52.8	1028	52.4
N	7048		3987		2198		1963	

Facilitators were gender matched staff from BLAST, MSC, and NM. They were selected on the basis of their experience and expertise. An intensive 14 day participatory training programme was arranged for the facilitators. BLAST staff with Bachelor’s degree in Law conducted sessions on laws and legal provisions. NM facilitators conducted training on gender and VAWG, while MSC engaged were fresh recruits with experience of SRHR intervention.

#### Community mobilisation campaigns

A 20-member community mobilization group was formed by NM in each SAFE site representing community leaders, local police, political leaders, NGO activists, and influential business owners in the locality for creating an enabling environment in the community for addressing VAW. Each group was provided a session on gender and VAWG subsequently about 11 short meetings were held with these support groups in each site.

NM recruited a total of 277 volunteers from all the study sites using the following criteria: leadership qualities, self-motivation for addressing VAWG, and rapport with the community. They were expected to foster positive change within and in their own communities in gender and VAWG related attitudes and practices. They received a day-long training based on SAFE’s Behaviour Change Communication (BCC) materials organized in five batches. Three one-day volunteer conventions were held during the project period. The volunteers distributed and discussed SAFE BCC materials with community members and VAWG survivors; linked the survivors with SAFE staff and services; and organized community campaigns.

Community campaigns applied methods developed by the Oxfam-led global initiative, We Can Campaign [28] and included celebration of gender related important events (e.g., International Women’s Day, 16 days of activism, etc) through poster distribution, billboard installation, wall painting, street drama, documentary film screening, concerts, banner campaign, reflective dialogue, etc. In total, 11 rallies, nine video shows, 11 folk music concerts, 11 mobile van campaigns, three quiz competitions, six reflective dialogue sessions, and eight banner campaigns were organized within and outside SAFE sites.

#### Health and legal service provision

SAFE’s “One-stop Service Centres” (OSC) located within or near MSCs were hubs for providing services and referrals; and disseminating information regarding other health/legal services. OSCs health services included: family planning, delivery, counselling and treatment of RTIs, STIs and HIV, and referrals. Legal services included counselling, mediation, representation, and referrals. The OSC staff received three-day training on gender, VAWG, SRHR, and legal provisions regarding SRH and VAWG prior to initiation of the intervention. The group members were informed of the services. SAFE staff referred community members requiring health or legal services to the OSCs. When required the women and girls were further referred by OSCs to other appropriate services (e.g., mental health counselling).

#### Training and advocacy

National level advocacy were conducted on gender and VAWG issues by BLAST, NM, and WCC. Training was conducted on gender, VAWG, and legal provisions with marriage registrars, police, lawyers, and the judiciary. SAFE’s media advocacy included a 12-episode live TV Talk Show covering gender issues, VAWG and SRHR.

### Intervention monitoring

Qualitative and quantitative data were routinely collected on implementation of the intervention. During fortnightly meetings of SAFE partners reports from qualitative and quantitative monitoring were presented and deviations, gaps, loopholes and challenges were discussed. Qualitative monitoring data were collected through 128 session observation, nine spot observation of OSCs, 10 event observation, 25 short In-Depth interviews with the group -members, eight Key-Informant interviews with service providers, four Focus Group Discussion with session facilitators, and six exit interviews of service receivers.

### Evaluation design

**Fig 2:**
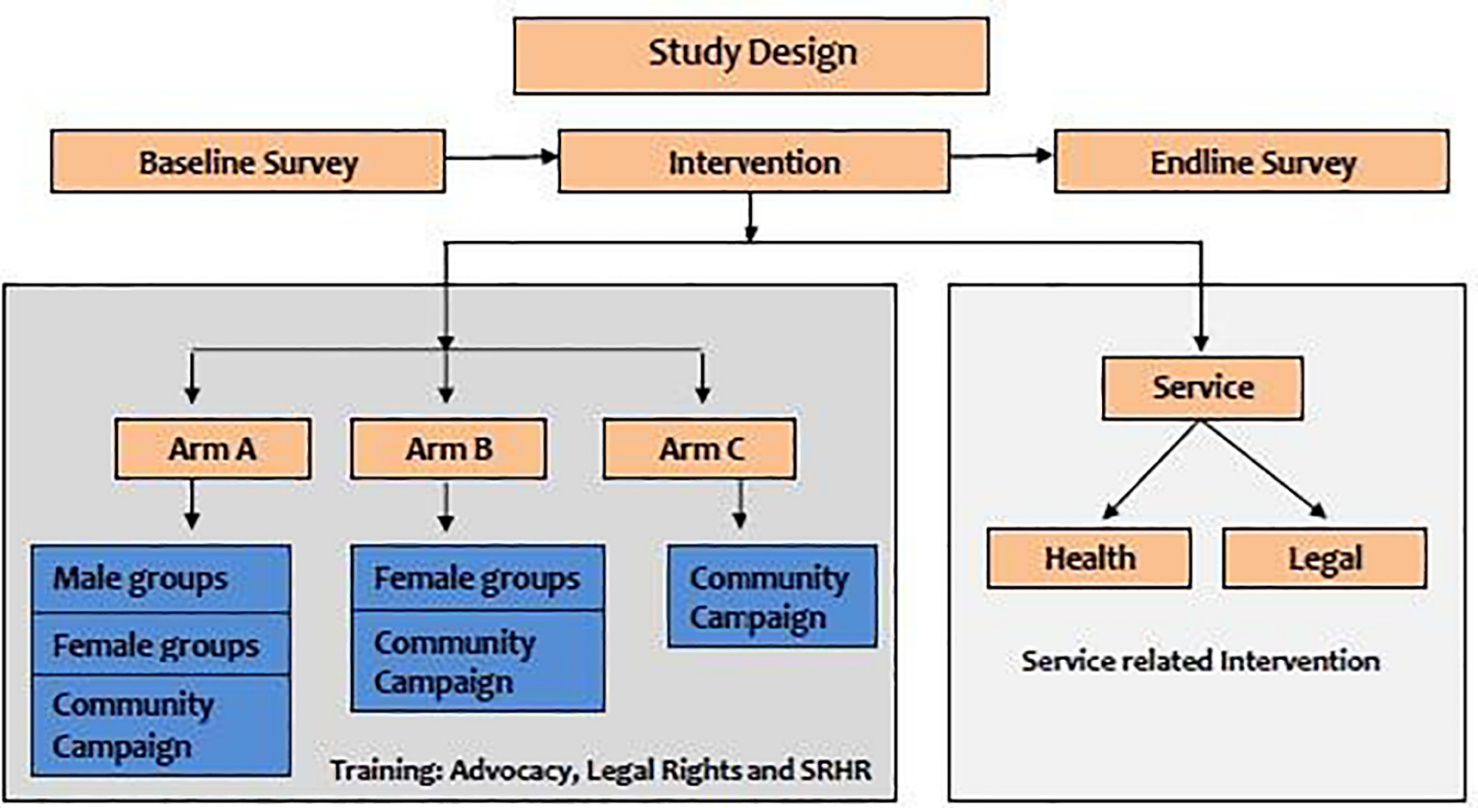
SAFE evaluation study design.

The SAFE evaluation used a three-arm multisite cluster RCT design, to test intervention strategies using blocking before randomizing clusters at the three study sites (Fig 2). The trial was registered at ClinicalTrials.gov and the registry number is NCT03280680 (https://clinicaltrials.gov/ct2/results?cond=&term=NCT03280680&cntry=&state=&city=&dist=). Arm A included community awareness-raising, OSC services and gender segregated sessions with female and male participants (C+F+M); Arm B included community awareness-raising, OSC services, and group sessions with only female participants (C+F); Arm C, the comparison arm, included community awareness-raising and OSC services, but had no group sessions (C).

SAFE evaluation aimed to test the intervention effect on (a) unmarried adolescent girls (aged 15–19 years), (b) married and unmarried females (aged 15–29 years), and (c) married and unmarried young men (aged 18–35 years). The study design was hierarchical with three fixed study sites, where *the clusters* were nested within study sites and the *individuals* were nested within clusters. SAFE clusters consisted of approximately 186 households each with or without natural boundaries. In order to reduce contamination, clusters were formed keeping buffer zones of 50–100 households or natural or infrastructural boundaries (e.g., water bodies or walls). Household enumeration and mapping were carried out to define clusters. The required number of clusters was achieved in nineteen slums. The clusters were randomly assigned to the three arms.

#### Sample size calculation

Different key outcome variables were considered for sample size calculation: (a) decreased rate of child marriage for adolescent girls, (b) decreased experience of violence in the past 18 months for young women, and (c) increased positive attitude regarding SRHR and VAWG for men, respectively. Accordingly, three different sample sizes were calculated based on different *minimum detectable effect sizes* (MDESs)– 55% for both young women and men [3], and 45% for adolescent girls. Using Optimal Design Software and considering 5% significance level, 80% power, intra-class correlation of 0.01 and a cluster size of 15 survey participants, the required number of clusters was 51 for adolescent girls and 27 for young women (overlapping with clusters of adolescents), and 27 for men per study site. Allowing for 20% over-sampling to address five percent non-response and 15% slum out-migration the cluster size was increased from 15 to 18. Thus, the final sample size corresponding to the different outcomes mentioned above were (a) 2,754 unmarried and married girls aged 15–19, (b) 1,458 females aged 20–29 regardless of marital status, and (c) 1,458 married and unmarried males aged 18–35. The sample size for unmarried adolescent girls was much higher due to a more conservative assumption about MDES. Given the amount of data, in this paper, we solely focus on the main IPV outcomes and a subgroup analysis separating out IPV against adolescent girls and young women.

#### Baseline and endline surveys

Baseline surveys were conducted prior to the intervention and endline surveys were conducted four months post intervention or 24 months post-baseline. Data were collected from 51 clusters of adolescent girls aged 15–19 years, 27 clusters of young women aged 20–29 years (overlapping with clusters of adolescents); and 27 clusters of 18–35 years old men. For ethical reasons female and male surveys were conducted in separate clusters. Sampling frame was obtained from enumeration data. The samples were drawn randomly. One study participant was selected randomly from each household. Interviews were conducted only after obtaining oral informed consent. The married minor females were considered as emancipated minors [34]. Thus, consent was obtained directly from them instead of assent from their guardians. Interviews were taken in a place convenient for the participant and suitable for avoiding interruptions. All interviews were conducted in private in a non-judgemental manner. The interviewers were gender-matched. Interviewers had Bachelor’s degree as minimum and were trained for 13 and 12 days respectively for the baseline and endline surveys.

Field experience showed a very high rate of unavailability of interviewees. Therefore, an additional 66% female sample and 118% male sample were drawn during baseline survey. Similarly, 37% female and 100% male sample were over-drawn at endline. The response rates calculated using the total number of finally targeted samples were: 64% for female survey at baseline and 80% at endline; and 51% at baseline and 56% at endline for male survey. At baseline, no interviews were conducted in the evening due to safety concerns. Based on feedback from intervention implementing partners interviews were also conducted in the evenings at endline. Thus, the endline interviews were conducted both early in the morning and in the evening, when the potential participants were available. This resulted in higher response rates at endline.

#### Measurement and analysis

The outcome in the present analyses is IPV in the last 12 months. It was measured using a modified version of the Conflict Tactics Scale (CTS) [35], which has been widely used worldwide, including many low and middle income countries and Bangladesh. The questionnaire explored physical, sexual, and economic violence perpetrated by a husband in the last 12 months using the following behaviourally explicit questions:

During the last 12 months has your most recent husband: (1) slapped or threw something at you that could hurt you? (2) pushed or shoved you? (3) hit you with a fist or with something else that could hurt you? (4) kicked you, dragged you, beat you up, choked you or burnt you intentionally? (5) threatened to use, or actually used, a gun, knife or other weapon against you?

The questions used for measuring sexual violence during the last 12 months were: (1) Did he physically force you to have sexual intercourse when you did not want to? (2) Did you ever have sexual intercourse that you did not want because you were afraid of what he might do? (3) Did he ever force you to do something sexual that you found degrading or humiliating?

Economic violence was measured using questions: During the last 12 months has your most recent husband: (1) prohibited you from getting a job, going to work, trading or earning money? (2) taken your money, gold, or any of your valuable things against your will? (3) thrown you out of the house? (4) kept money from his earnings for alcohol, drug, gambling, tobacco, or other things for his own use when he knew you were struggling to afford household expenses?

The whole questionnaire was pre-tested prior to training of the survey team. Piloting of the questionnaire was conducted in slums outside the study area at the end of the training. The initial questionnaire underwent modifications based on pre-testing and piloting.

The response options for the items of physical, sexual, and economic violence were coded as “Yes = 1” and “No = 0”. Prevalence of each was calculated as proportion of currently married women who reported exposure to any act of that specific form of violence in the last 12 months.

Descriptive analysis was performed to report frequencies of different forms of violence. Chi-square and t-tests were performed to check whether the study arms were covariate balanced in each survey and across surveys by arm and gender. SAFE’s impact was assessed using risk ratios derived from binary regression analyses for measuring change in outcomes in the intervention arms relative to change in the comparison arm. Mixed effect models were constructed with cluster as random effect and survey, intervention group, and survey×intervention interaction as fixed effects. Adjusting for IPV at baseline survey gave us a measure of the relative change in intervention group that took place between the baseline and the endline compared to the change in the comparison group.

Currently married women aged 15–29 years (n = 2,666 at baseline and n = 2,670 at endline) and currently married men aged 18–35 (n = 930 at baseline and n = 1,026 at endline) were included in the analyses. Although the required sample size for IPV analyses was 2,430 the total sample size achieved was 5,336 for currently married women aged 15–29 for fulfilling sample requirement for measuring SAFE’s effect on child marriage.

Background characteristics of adolescent girls and young women were statistically significant differences, which made us conduct sub-group analyses. There is no significant difference in prevalence of IPV between adolescent girls and young women in Bangladeshi slums [36]. So, the required sample size was same for the two groups (2,430). The achieved married adolescent sample size was 2,463 enabling us to measure SAFE’s effect on IPV against adolescent girls. The female data were adjusted using age-specific weights. Since the study used a multisite cluster RCT all regression analyses performed adjusted for clusters. Additionally, the covariates which differed significantly between baseline and endline surveys (female age, education and working status) were adjusted. Significance level was set at p < .05 for all descriptive and regression analyses. Statistical analyses were performed using STATA version 13.

### Ethical issues

The study followed recommendations for researching VAWG [37–38]. The study was approved by Institutional Review Boards (IRB) of icddr,b (PR#10007) and Population Council (PR#507). As mentioned above, oral informed consent was obtained before interviews. Written consent was not used due to low levels of literacy and concerns regarding confidentiality. All the participants were informed orally of the purpose and nature of the study, its expected benefits, sensitivity, confidentiality and voluntary nature of participation in front of the supervisor, who served as the witness and signed the consent form in that capacity. Interviews were conducted in a place convenient for the participant (at home in most cases); in private; and in a non-judgmental way. Interviews were rescheduled or conducted in multiple sessions according to participants’ availability or when privacy could not be maintained. Name and addresses of the study participants were strictly confidential and were not entered in the data file. De-identified data were analyzed and presented.

## Results

### Background characteristics of the survey samples

Table 2 presents background characteristics of the currently married female samples by arms and baseline and endline surveys. Three types of comparisons were made for statistical significance: (1) between arms at baseline, (2) between arms at endline, and (3) between surveys in each arm.

**Table 2:** Background characteristics of currently married women aged 15–29 years by arm and by survey.

Background characteristics^a^	Baseline			Endline		
	Community + Female + Male	Community + Female	Community	Community + Female + Male	Community + Female	Community
N	879	905	882	866	918	886
Mean age (SD)	22·9 (3·4)	22·8 (3·4)	23·0 (3·4)	22·9 (3·3)	23·2 (3·5)^2^	23·1 (3·4)^3^
Age group, %						
15–19 years	16·4	17·8	16·6	18·0	17·8	16·7
20–29 years	83·6	82·2	83·4	82·0	82·2	83·3
Mean education (SD)	3·8 (3·3)	4·0 (3·3)	4·4 (3·5)	4·5 (3·3)^1^	4·3 (3·3)^2^	4·7 (3·4)^3^
Education group,%						
No education	29·6	29·1	24·5	21·4	22·7	18·5
Primary	45·3	40·4	41·6	46·1	46·2	44·1
Secondary or higher	25·1	30·6	33·9	32·6^1^	31·1^2^	37·4^3^
Muslim	98·9	99·5	98·9	98·6	99·3	98·9
Ever worked,%	59·2	57·4	52·4	67·5^1^	66·5^2^	64·2^3^
Safe member, %	-	-	-	4·7	2·8	0·9

^a^ All the percentages and means are weighted

^1^ Statistically significant difference between Community+Female+Male Arms at baseline and endline at 5% level

^2^ Statistically significant difference between Community+Female Arms at baseline and endline at 5% level

^3^ Statistically significant difference between Community Arms at baseline and endline at 5% level

Background characteristics of the samples had no statistically significant differences across the three arms either at baseline or endline. Hence, the arms were covariate balanced. However, in each arm, there were significant differences in age, education, and employment history of females between baseline and endline. Therefore, the risk ratio analyses were adjusted for age, education and employment history. The whole sample was predominantly Muslim (99–100%).

The married adolescent and young women samples had statistically significant differences in both surveys (Table 3). The married adolescent sample was relatively more educated. Duration of marriage was about 3–3·5 times higher among the young women compared to the adolescent girls. Approximately 89% of the young women had at least one child, whereas this proportion varied between 41% and 47% among the adolescents. In both baseline and endline surveys, a lower proportion of the adolescents ever worked compared to the young women sample (53% and 56% at baseline; and 57% and 68% at endline). The spouses of the adolescent girls were younger and more educated than the spouses of young women. The difference between mean age of successfully interviewed and not interviewed (sampled but could not be interviewed) ever-married samples was below one year at baseline and endline surveys (Table 4). However, due to large sample size these differences were statistically significant for baseline in the young women sample in both surveys. Hence, the results of regressions were age adjusted.

**Table 3:** Background characteristics of currently married adolescent girls and young women at baseline and endline.

Characteristics***	Baseline (n = 2666)		Endline (n = 2670)	
	Adolescent girls aged 15–19 (n = 1,277)	Young women aged 20–29 (n = 1,389)	Adolescent girls aged 15–19 (n = 1,186)	Young women aged 20–29 (n = 1,484)
Education, mean, year	4·4	4·0	5·2	4·4
Education, %				
No education	19·5	29·4	12·3	22·7
Grade 1–4	29·5	26·6	25·4	26·2
Grade 5	16·5	15·1	19·8	19·3
Grade 6–9	30·9	22·7	35·1	25·1
Grade 10 or above	3·6	6·2	7·4	6·7
Ever worked*, %	53·2	56·9	56·1	68·1
NGO membership, %	1·3	3·1	3·4	11·0
Marriage duration, year	2·5	8·3	2·4	8·4
Have at least one child, %	46·5	89·2	40·6	88·7
SAFE group membership, %	-	-	2.2	3.7
Husband’s age, mean, year	24·6	30·6	24·3	30·6
Husband’s age, %				
16–25	71·6	14·9	71·9	13·0
26–60	28·5	85·0	28·1	87·0
Husband’s education, mean, year	5·1	4·6	5·7	4·9
Husband’s education, %				
No education	20·7	29·1	15·6	25·9
Grade 1–4	12·1	11·4	13·9	14·0
Grade 5	17·2	14·7	19·1	18·4
Grade 6–9	28·2	22·0	32·8	25·6
Grade 10 or above	9·2	11·4	14·1	12·9
Don’t know	12·6	11·5	4·4	3·2

***All background characteristics shown differ significantly between adolescent girls and young women at baseline and at endline at 1% level

* Only exception was found here. The difference of ever working status was not significant at baseline at 1% level but was significant at 10% level.

**Table 4:** Background characteristics of sample successfully interviewed and not interviewed, SAFE surveys.

	Baseline			Endline		
	Successful cases	Unsuccessful cases	p-value	Successful cases	Unsuccessful cases	p-value
Ever married adolescent girls aged 15–19 years						
N	1485	398		1329	389	
Mean age (SD)	17·89 (1·11)	17·97 (1·00)	·213	17·87 (1·11)	17·98 (·98)	0·091
Ever married young women aged 20–29 years						
N	1504	752		1621	308	·1124
Mean age (SD)	23·69 (2·75)	24·07 (2·68)	·0016***	23·91 (2·77)	24·18 (2·72)	

***p < .01

### Impact of SAFE

#### Impact of SAFE on spousal violence against females aged 15–29

As shown in Table 5, IPV prevalence was very high both at baseline and endline. However, a lower proportion of the females in each arm reported physical, sexual, economic, and emotional IPV at endline compared to baseline. Thus, prevalence of physical IPV was reduced by 9–14%. Reduction of sexual, economic, and emotional IPV from baseline to endline was between 17–21%, between 17–23% and between 15–18% respectively. Regression analyses showed no statistically significant impact of SAFE on IPV against women aged 15–29 as reported by females (Table 5) and males (results not shown).

**Table 5:** Impact of SAFE intervention on spousal violence against currently married women in Dhaka slums.

Indicator and intervention arm	Change over time, %			Adjusted^c^ RR (95% CI)	
	Before (%)	After (%)	After–Before (%)	Impact of female group [(C+F)-C = F]^a^	Impact of female and male groups [(C+F+M)-C = (F+M)]^a^
**Spousal violence against currently married women aged 15–29**^b^^,^ ^c^ (baseline n = 2,666; endline n = 2670)					
**Any physical violence**					
A. Community + Female + Male	58·7	49·5	-9·2	1·05 (·90–1·22)	1.09 (·93–1·28)
B. Community + Female	58·7	48·1	-10·6		
C. Community	58·0	44·5	-13·5		
**Any sexual violence**					
A. Community + Female + Male	59·2	38·6	-20·6	·96 (·80–1·14)	·96 (·79–1·18)
B. Community + Female	59·3	39·3	-20·0		
C. Community	55·5	38·5	-17·0		
**Any economic violence**					
A. Community + Female + Male	50·6	30·4	-20·2	·88 (·70–1. ·10)	1·01 (·84–1·20)
B. Community + Female	48·2	25·6	-22·6		
C. Community	43·4	26·3	-17·1		
**Any emotional violence**					
A. Community + Female + Male	66·5	51·2	-15·3	·96 (·77–1·22)	1·04 (·85–1·28)
B. Community + Female	64·8	46·8	-18·0		
C. Community	61·9	46·0	-15·9		
**Spousal violence against currently married adolescent girls aged 15–19**^**b**^^,^ ^**c**^ (baseline n = 1,277; endline n = 1186)					
**Any physical violence**					
A. Community + Female + Male	59·5	40·3	-19·2	·88 (·71–1·09)	·79 (·62-·99)**
B. Community + Female	60·0	45·0	-15·0		
C. Community	57·2	47·7	-9·5		
**Any sexual violence**					
A. Community + Female + Male	57·9	34·0	-23·9	·97 (·77–1·21)	·85 (·65–1·09)
B. Community + Female	58·1	38·7	-19·4		
C. Community	53·9	37·4	-16·5		
**Any economic violence**					
A. Community + Female + Male	54·2	30·1	-24·1	1·21 (·92–1·60)	1·04 (·81–1·33)
B. Community + Female	51·1	35·2	-15·9		
C. Community	56·8	30·9	-25·9		
**Any emotional violence**					
A. Community + Female + Male	64·7	36·4	-28·3	·92 (·69–1·22)	·81 (·61–1·08)
B. Community + Female	64·0	40·1	-23·9		
C. Community	59·6	41·0	-18·6		
**Spousal violence against currently married young women aged 20–29**^**b**^^,^ ^**c**^ (baseline n = 1,389; endline n = 1,484)					
**Any physical violence**					
A. Community + Female + Male	58·5	51·6	-6·9	1·09 (·92–1·29)	1·16 (·97–1·39)
B. Community + Female	58·4	48·8	-9·6		
C. Community	58·2	43·9	-14·3		
**Any sexual violence**					
A. Community + Female + Male	59·5	39·6	-19·9	·96 (·77–1·18)	·99 (·79–1·24)
B. Community + Female	59·5	39·4	-20·1		
C. Community	55·8	38·8	-17·0		
**Any economic violence**					
A. Community + Female + Male	49·8	30·5	-19·3	·79 (·58–1·07)	·98 (·77–1·25)
B. Community + Female	47·5	23·6	-23·9		
C. Community	40·7	25·4	-15·3		
**Any emotional violence**					
A. Community + Female + Male	66·8	54·5	-12·3	·97 (·74–1·27)	1·08 (·84–1·39)
B. Community + Female	65·0	48·3	-16·7		
C. Community	62·3	47·0	-15·3		

^**a**^ C = Community, F = Female group, M = Male group

^b^ Weighted percentage

^c^ Adjusted for age, education and ever working status

**p < .05

#### Impact of SAFE on spousal violence against adolescent girls and young women

Subgroup analyses of adolescent girls (aged 15–19) and young women (aged 20–29) showed that SAFE significantly lowered the risk of experiencing physical IPV among adolescent girls in the community (aRR 0·79; 95% CI 0·62, 0·99) (Table 5). Risk of sexual IPV exposure was consistently lower in all age groups and arms. However, the results did not achieve statistical significance.

## Discussion

There are still few RCTs measuring intervention impact on IPV in developing countries and even fewer measuring such impact at the community-level. SAFE is the first RCT in South Asia and Bangladesh assessing impact of an intervention on IPV in the community. It measured effectiveness of female group intervention and gender segregated female and male group intervention and activism of the group members on IPV in the community. The main results show that none of the SAFE intervention strategies had any effect on IPV against females aged 15–29 refuting our hypotheses.

Subgroup analyses, however, demonstrate that M+F intervention is more effective than F only intervention against adolescent girls in the community. Our hypothesis that female only intervention would reduce IPV was refuted. Reduction in physical IPV against adolescent girls by 21% is an important achievement as the literature repeatedly indicates that the adolescents are more vulnerable to IPV than older women [39–40]. This finding highlights benefit of targeting men on top of women in IPV prevention efforts, which has also been illustrated by other researchers [1].

Young age, higher levels of education, shorter duration of marriage, and relatively new experiences of IPV may have made the adolescent girls more proactive in dealing with physical IPV, contributing to its reduction. Due to shorter duration of marriage in which practices of violence that had not yet been “normalized” in a relationship may also have been easier to address. Literature indicates that young men are amenable to change and that they can become allies of movements for eliminating VAWG in different settings [33]. Lower age and higher education of the adolescent girls’ husbands compared to young women’s husbands may have contributed to reduction of physical IPV among adolescent girls as these characteristics are recognised to accompany openness to new ideas [39,41–42].

Questions may arise as to why SAFE did not achieve a greater magnitude of impact similar to some other studies such as the IMAGE, where a 55% reduction in IPV was demonstrated among group members [3]. We argue that effect size of IMAGE is not comparable to that of SAFE as the first measured effect among group members and the second–among a representative sample of the cluster resulting in 1–5% of the sample being SAFE group members.

Intervention coverage may also have contributed to smaller effect size and inability of SAFE in reducing other types of violence. As mentioned above 46% of females aged 15–29 and only 15% of males aged 18–35 were SAFE group members. Further research is required for understanding whether higher coverage of the males would make IPV prevention more effective.

SAFE’s achievement of community level impact through group sessions conducted with part of the eligible community members highlights success of diffusion in this intervention. These results also underline that IPV is an extremely complex issue demanding different strategies for addressing different manifestations of it in different groups of women even in the same context. These findings raise more questions than they answer highlighting the need for more research to understand why impact of SAFE differed by age groups of females and what would work in reducing the forms of IPV that remained untouched by SAFE.

### Limitations

It is possible that social desirability bias may make the female sample under report IPV in the intervention arms [43]. However, greater awareness about IPV in the context of silence around IPV is expected to reduce under reporting rather than increasing it [3]. Contamination may be an issue in SAFE, which would lead to underestimation of the effect. This study did not allow measurement of such possible underestimation. Due to high mobility among slum population, it was not possible to measure SAFE’s effect on closed cohort of group members. The control group was not a true control and received community mobilization and services. Moreover, due to given project timeline this evaluation has captured a relatively short-term (4 month) effect of SAFE, whereas both SHARE and Stepping Stones found intervention effect at a much later point in time [5–6].

Despite these limitations, SAFE were successful in demonstrating an effect at the community level. The only other intervention that had community level effect was SHARE in Uganda [6]. It is worth noting that while SHARE achieved such effect mainly through community mobilization; SAFE achieved it through interactive group sessions with young women; and with adolescent girls and young men in their communities, where promotion of activism was an important component. More research is needed to understand the pathways through which such changes occur.

### Conclusions

SAFE makes important contribution to the literature by showing the benefit of targeting both women and men for addressing spousal violence against adolescent girls. While several RCTs in Africa combining economic empowerment of women with gender sensitisation proved successful in reducing IPV (IMAGE, Cote De Voir, Creating Futures) SAFE proves that in an impoverished setting with some female employment opportunities gender sensitisation of females and males may reduce physical IPV against adolescent girls, which makes it easily scalable. However, it is essential for analysing prospects of sustainability and cost of interventions such as SAFE before decisions can be made about scaling it up. More research is warranted for understanding what works in addressing forms of IPV that SAFE did not reduce. The findings highlight the need for tailoring different interventions for different age groups of females in a patriarchal setting. This intervention needs to be replicated in similar settings for confirming its effectiveness.
